# Fungal Biodiversity From the Atlantic Forest With Bioactive Metabolites Against Cutaneous Leishmaniasis

**DOI:** 10.1002/cbdv.202501468

**Published:** 2025-08-28

**Authors:** Juliana Cristina Pereira Calado, Marina Valente Navarro, Alison Felipe Alencar Chaves, Isis Casula, Patricia Locosque Ramos, João Batista da Cruz, Anderson Messias Rodrigues, Keith Dayane Leite Lira, Fernando Luiz Affonso Fonseca, Patricia Xander, Suzan Pantaroto de Vasconcellos, Wagner Luiz Batista

**Affiliations:** ^1^ Department of Microbiology, Immunology and Parasitology Federal University of São Paulo Sao Paulo Brazil; ^2^ Secretariat of the Environment of the State of São Paulo Sao Paulo Brazil; ^3^ Department of Pharmaceutical Sciences Federal University of São Paulo Sao Paulo Brazil

**Keywords:** biodiversity, bioactive compounds, *Leishmania* (*Leishmania*) *amazonensis*

## Abstract

Biodiversity offers a rich source of bioactive compounds for drug discovery. We isolated *Aspergillus fumigatus* from compost in the Atlantic Forest and found its extract active against *Leishmania (Leishmania) amazonensis*. Two crude extracts, I06 and I75, were selected for further study. I06 reduced parasite burden and infection rates in human (THP‐1) and murine (BMDM) macrophages, with a half‐maximal inhibitory concentration of 1 µg/mL, similar to the reference drug pentamidine. Liquid chromatography–mass spectrometry identified extract components. I06‐treated parasites showed morphological changes, Annexin V labeling, and reduced mitochondrial membrane potential, indicating late apoptosis. Reactive oxygen species increased within 15 min of I06 exposure, faster than pentamidine. I06 was non‐hemolytic in vitro and in vivo, and intraperitoneal treatment reduced lesion size and parasite load in mice. These findings support I06 as a promising candidate for leishmaniasis therapy and highlight microbial biodiversity as a valuable source of novel bioactive compounds.

## Graphical abstract

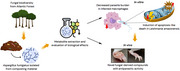

## 1 Introductions

Fungi are a diverse group of microorganisms with a remarkable ability to synthesize a multitude of natural products, many of which are essential for their survival [1]. These natural products, commonly known as secondary metabolites (SMs), are produced via complex metabolic pathways originating from primary metabolite pools [2]. SMs are synthesized and released into the extracellular environment during fungal growth, where they serve as crucial signaling molecules that initiate fungal development and maintain cellular structures and functions [3]. It is important to note that only a subset of SMs exhibit bioactivity, making them particularly relevant for drug discovery and pharmaceutical applications. The loss or alteration of genes involved in SM biosynthesis can lead to the formation of aberrant fungi incapable of producing specific metabolites.[4] As a result, understanding the biosynthesis and function of SMs in fungi is critical for deepening our knowledge of fungal biology and exploring their potential applications in various fields.

The genes responsible for the biosynthesis of SMs are typically located in gene clusters and are transcribed and translated into enzymes that act in concert to produce these complex biomolecules [5, 6]. In filamentous fungi, the gene clusters are highly intricate and can generate an extensive number of combinations to produce SMs [2]. The biosynthesis of SMs in fungi is regulated by various factors, including transcriptional and epigenetic activation, which are in turn modulated by environmental stimuli and the fungal growth stage [7, 8]. The ability of fungi to produce a diverse array of SMs results from these complex regulatory mechanisms, enabling them to synthesize different molecules depending on prevailing environmental conditions [9, 10]. This remarkable adaptability allows fungi to thrive in diverse environments and underscores their potential as a source of novel natural products with a broad range of biological activities.

Moreover, the metabolic diversity of microorganisms, including fungi, supports the production of a vast array of compounds with diverse biological activities [11]. Many of these bioactive molecules have demonstrated enzymatic [12], antibiotic [13], antitumor [14], and antiparasitic [15] activities, as reported in scientific literature. Certain environments, such as the Atlantic Forest in Brazil, are known to harbor exceptional biodiversity, potentially enhancing the capacity of microorganisms to produce bioactive compounds [16, 17]. The Atlantic Forest is considered one of the richest floras on the planet, with at least 50 000 species, representing one‐sixth of the total global flora [18]. The identification and characterization of bioactive compounds from diverse microbial sources, including fungi, have emerged as key topics in drug discovery and pharmaceutical development. Therefore, the exploration of microorganisms from unique ecological niches, such as the Atlantic Forest, may provide novel sources of natural products with therapeutic potential and represent a promising avenue for advancing biomedical research.

Composting is a well‐known source of microorganisms with remarkable biological activity, as previously described in studies conducted at the São Paulo Zoo Park Foundation (SPZPF) composting facility [19]. Composting is a biological process involving the breakdown of organic matter by microorganisms such as bacteria, fungi, and actinomycetes [20, 21]. The unique combination of microorganisms in composting, combined with the biodiversity of the Atlantic Forest, has the potential to promote diversification and interaction within the microbial community, leading to the production of novel compounds with biological activity. Environmental changes, including those associated with the composting process, have been shown to influence the production of SMs in fungi [8]. Therefore, exploring the impact of such changes on the microbial community and the resulting production of bioactive compounds is crucial for identifying new sources of natural products with potential biomedical applications. The integration of composting and Atlantic Forest biodiversity represents a promising strategy for discovering novel bioactive compounds and advancing our understanding of the ecological and molecular mechanisms that govern microbial community dynamics and natural product biosynthesis.

Leishmaniasis, a neglected tropical disease prevalent in many developing countries, is caused by protozoan parasites of the genus *Leishmania*. Leishmaniasis is endemic in 99 countries, affecting more than 1 billion people living in endemic areas, with around 1 million cutaneous cases occurring annually [22]. Therefore, the discovery of new, effective antiparasitic agents is of great importance. Current treatments for leishmaniasis are unsatisfactory, and there is an urgent need for new therapeutic strategies to reduce its spread. Despite the availability of several treatment options, including chemotherapy and immunotherapy, these approaches have limitations such as high toxicity, low efficacy, and the emergence of drug‐resistant strains [23]. Recent advances in the understanding of the pathogenesis of leishmaniasis, combined with the growing availability of natural product libraries and high‐throughput screening technologies, have facilitated the identification of several promising lead compounds with anti‐leishmanial activity [24].

Our study aimed to evaluate the antiparasitic activity of fungal SMs against *Leishmania (Leishmania) amazonensis* both in vitro and in vivo. Among the SMs produced by an *A. fumigatus* isolate sourced from composting in the Atlantic Forest, we identified promising compounds with potent antiparasitic activity against *L. (L.) amazonensis*. Our study included in vitro assays to evaluate the activity of these compounds against the parasite, as well *as* in vivo *studies* to assess their efficacy and safety in a mouse model of leishmaniasis. Overall, our results highlighted the potential of *A. fumigatus* as a source of novel antiparasitic agents and underscore the importance of exploring the unique ecological niches of microorganisms for drug discovery.

## 2 Results and Discussion

### 2.1 Anti‐Leishmanial Activity of Fungal SMs In Vitro

Fungal isolates were obtained from the composting facility of the São Paulo Zoo, which processes organic plant material derived from the Atlantic Forest biome. These fungi were isolated from compost with the aim of evaluating the biological activity of their SMs. Fungal SMs were incubated with promastigote forms at various concentrations (ranging from 80 to 2.5 µg/mL) for 48 h. Among the 71 fungal isolates screened, 12 produced compounds with significant biological activities against promastigotes of *Leishmania (L.) amazonensi*s, exhibiting half‐maximal inhibitory concentration (IC_50_) values lower than 5 µg/mL (Table 1).

**TABLE 1:** Leishmanicidal activity against the promastigote form of *Leishmania (L.) amazonensis (*in vitro*)*.

sample	ic_50_ (µg /mL)	
	*L*. (*L*.) *amazonensis*	Cell line 3T3
**i05**	4.79	3.54
**i06**	0.98	49.72
**i26**	4.78	22.62
**i35**	3.25	1.47
**i37**	4.83	3.10
**i48**	2.45	1.43
**i49**	2.37	1.33
**i50**	4.68	2.43
**i57**	2.89	1.24
**i67**	4.66	1.73
**i68**	2.39	1.09
**i75**	3.79	21.88

The selectivity index (SI) was calculated as the ratio between the CC_50_ for 3T3 fibroblasts and the IC_50_ for protozoa, providing a measure of parasite selectivity over host cell toxicity. Extracts from isolates I06, I26, and I75 exhibited SI values of 50.7, 4.7, and 5.8, respectively. This indicates that the I06 extract was 50.7 times more selective for parasites compared to host cells. All three isolates were identified as *Aspergillus fumigatus* based on sequence analysis of the ITS region and the calmodulin gene (Table 2).

**TABLE 2:** Identification of fungal isolates by sequencing of the ITS region and calmodulin gene.

Fungal isolate	ID ITS	Identity (%)	Access number
I06	*A. fumigatus*	99	KY827337.1
I26	*A. fumigatus*	100	KY827337.1
I75	*A. fumigatus*	99	MG674824.1

**FIGURE 1:**
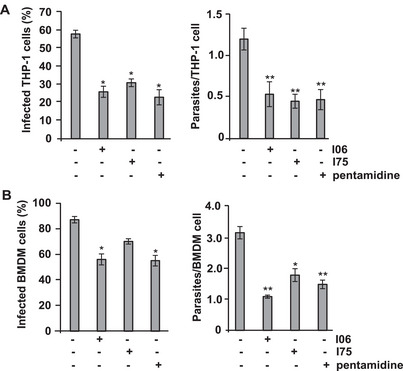
Leishmanicidal activity of secondary metabolites (SMs) in vitro against amastigote *L. (L.) amazonensis* in THP‐1 cells and bone marrow‐derived macrophages. (A) THP‐1 cells (2x10^5^) were differentiated into macrophages with 50 ng/mL of phorbol 12‐myristate 13‐acetate (PMA) for 24 h. Then were incubated with 2 × 10^6^ parasites/mL for 2 h and treated with the half‐maximal inhibitory concentration (IC_50_) SMs I06 (1 µg/mL) or I75 (3.8 µg/mL) for 24 h. (B) Bone marrow‐derived macrophages from BALB/c were incubated with 2 × 10^6^ parasites/mL for 2 h and treated with IC_50_ SMs I06 (1 µg/mL) or I75 (3.8 µg/mL) for 24 h. The quantity of parasites and infected cells was counted in 100 cells. Pentamidine (10 µg/mL) was used as a control. Data are presented as mean ± SD (*n* = 3 independent experiments). Statistical analysis was performed using one‐way analysis of variance (ANOVA) followed by Student's *t*‐test, two‐tailed, α = 0.05, after confirmation of normal distribution and homogeneity of variances. Symbols indicate significance: * *p* ≤ 0.05; ** *p* ≤ 0.01. Analyses were performed using GraphPad Prism 6 (GraphPad Software Inc.).

The anti‐leishmanial potential of I06 and I75 extracts was further evaluated in vitro infection models using PMA‐differentiated THP‐1 macrophages and murine bone marrow‐derived macrophages (BMDMs) infected with *L. (L.) amazonensis* amastigotes. In THP‐1 cells, the infection ratio (IR) ranged from 0.7 to 0.8 at a multiplicity of infection (MOI) of 1:10. Treatment with I06 and I75 extracts resulted in a significant reduction in IR values (Figure 1A), comparable to those observed with pentamidine treatment. In untreated THP‐1 cells, the average parasite burden was 1.3 amastigotes per cell (Figure 1A, right panel). Treatment with I06 (1 µg/mL) or I75 (3.8 µg/mL) extracts led to a 2.4‐fold decrease in the number of parasites per cell.

In mouse BMDM cells infected with *L*. (*L*.) *amazonensis* amastigotes, the IR was 0.9. Treatment with I06 or I75 extracts reduced the IR to 0.56 or 0.7, respectively (Figure 1B). The IR value of the I06 sample was comparable to that observed for pentamidine (0.57), and the average number of parasites per cell in I06 and I75 samples was 2.8‐fold and 1.5‐fold lower, respectively, compared with control cells (Figure 1B, right panel). These findings suggest that both I06 and I75 contain compounds with potent leishmanicidal activity against both promastigote and amastigote forms of *L*. (*L*.) *amazonensis*. Notably, the I06 extract demonstrated high selectivity. These findings highlight that the SMs from *A. fumigatus* isolates recovered from composted plant material in a biodiverse Atlantic Forest biome demonstrated promising antileishmanial activity. Although *A. fumigatus* is recognized as an opportunistic pathogen, the environmental strains may display unique metabolic profiles shaped by selective pressures in their natural habitat. Nevertheless, the inherent pathogenic potential of *A. fumigatus* warrants strict biosafety precautions and careful phylogenetic characterization before any translational application.

### 2.2 I06 Extract Induces Apoptosis‐like in *L*. (*L*.) *amazonensis*

**FIGURE 2:**
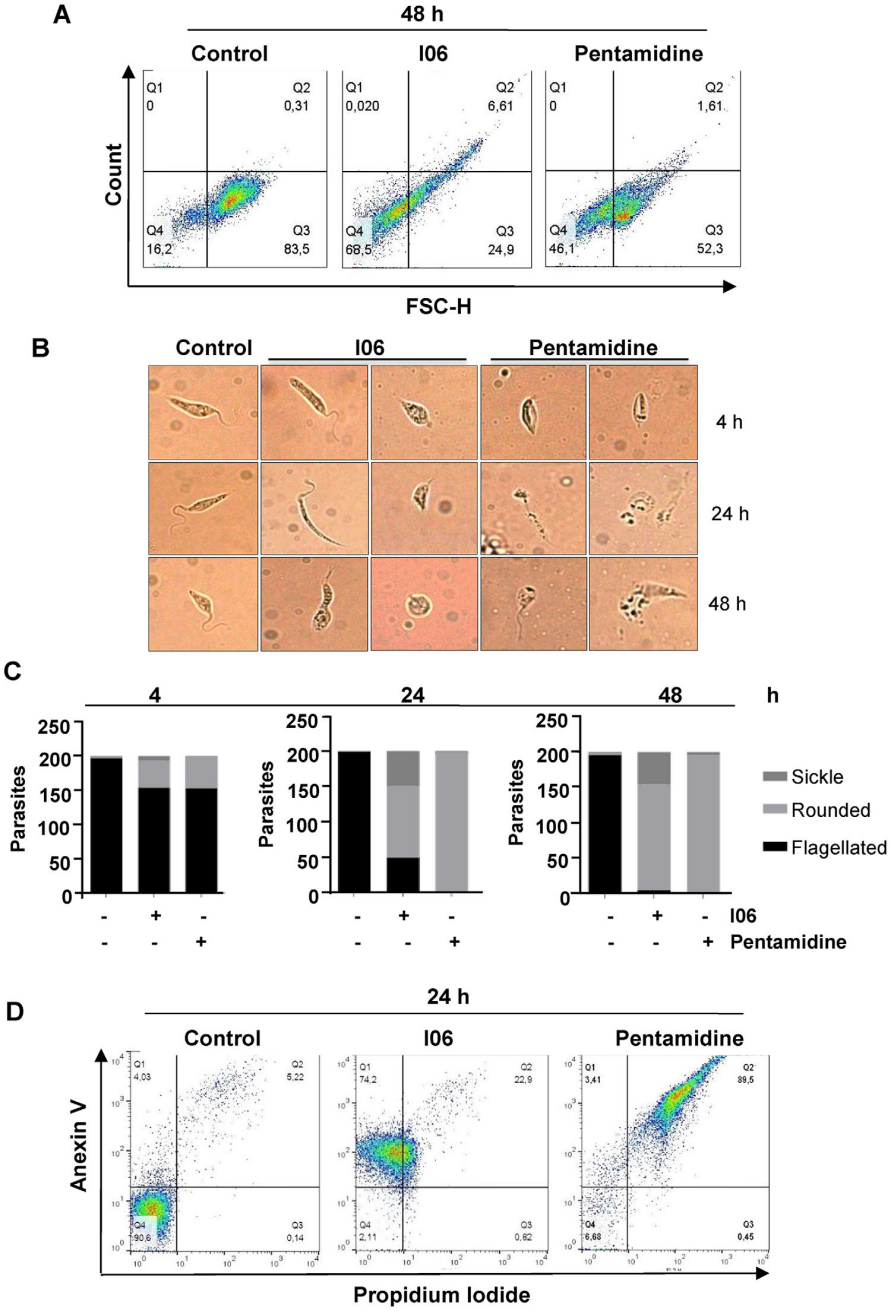
**Secondary metabolite (SM) I06 alters promastigote size and induces apoptosis**. (A) Promastigotes were incubated with SM I06 (1 µg/mL) for 48 h and analyzed by flow cytometry. Dot plots represent changes in cell size. (B) Morphological analysis of *L. (L.) amazonensis* promastigotes treated with SM I06. Parasites (1 × 10⁶) were incubated with SM I06 (1 µg/mL) for 4, 24, and 48 h, and examined by optical microscopy (100× magnification). Pentamidine (10 µg/mL) was used as a positive control. (C) Quantification of promastigote morphology. A total of 200 parasites were evaluated by microscopy and classified into three morphological categories: sickle‐shaped, rounded, and flagellated forms. (D) Induction of apoptosis by SM I06. Promastigotes (1 × 10⁶) were treated with SM I06 (1 µg/mL) for 24 h, stained with Annexin V and propidium iodide (PI), and analyzed by flow cytometry. Pentamidine (10 µg/mL) was used as a control. Data are presented as mean ± SD (*n* = 3 independent experiments).

During the death process of *Leishmania* promastigotes, cellular morphology typically shifts from a flagellated to a rounded shape, reflecting a reduction in size [25]. To assess whether the I06 extract induces such changes, we evaluated promastigote morphology by flow cytometry. After 48 h of exposure to the extract, 68.5% of the parasite population exhibited reduced size compared to untreated controls (Figure 2A). Phase‐contrast microscopy revealed three distinct morphotypes among treated parasites: flagellated, rounded, and thinned forms (Figure 2B). We next examined morphological alterations in promastigotes treated with the IC_50_ concentration of the I06 extract for 24 and 48 h. Treatment with 1 µg/mL led to pronounced reductions in cell size and shape, as well as marked loss of flagella, when compared to untreated controls (Figure 2C). In contrast, promastigotes treated with pentamidine (10 µg/mL) displayed complete rounding, accompanied by cytoplasmic condensation and shrinkage (Figure 2C). Collectively, these findings suggest that the I06 extract induces significant apoptotic‐like morphological changes in *L*. (*L*.) *amazonensis* promastigotes.

Given that morphological alterations are key indicators distinguishing apoptotic from necrotic cell death [26], we investigated the type of death induced by the I06 extract. After 24 h of treatment, parasites showed significant increases in phosphatidylserine exposure (*p* ≤ 0.05) and loss of plasma membrane integrity (*p* ≤ 0.05) (Figure 2D). Annexin V/propidium iodide (PI) labeling revealed that 74.2% of treated parasites were Annexin V^+^/PI^‐^ (early apoptosis‐like), 22.9% were Annexin V^+^PI^+^ (late apoptosis‐like), 0.82% were Annexin V^‐^/PI^+^ (necrotic), and only 2.1% remained viable (Annexin V PI^‐^). By contrast, 90.6% of control parasites were viable (Figure 2D). Under these conditions, pentamidine treatment produced a labeling consistent with necroptosis (Figure 2D).

### 2.3 I06 Extract Induces Mitochondrial Dysfunction by Depolarizing the Mitochondrial Membrane Potential (*Δψ_m_*) and Increasing Reactive Oxygen Species Production in *L*. (*L*.) *amazonensis*

**FIGURE 3:**
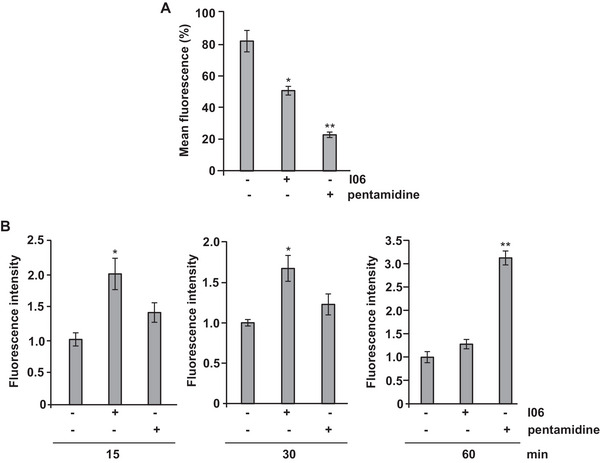
**Secondary metabolite (SM) I06 decreases the potential of mitochondria (Δ*Ψ*_m_) and increases the reactive oxygen species (ROS) production**. (A) Measurement of mitochondrial membrane potential using Rhodamine 123 following 24 h of SM I06 (1 µg/mL) treatment in *L*. (*L*.) *amazonensis* promastigotes (1 × 10^6^). (B) 2’,7’‐Dichlorodihydrofluorescein diacetate (H_2_DCF‐DA) assay performed for detection of ROS generation after treatment with SM I06 (1 µg/mL) for 15, 30, and 60 min in *L*. (*L*.) *amazonensis* promastigotes (1 × 10^6^). Pentamidine (10 µg/mL) was used as a control. Data are presented as mean ± SD (*n* = 3 independent experiments). Statistical analysis was performed using one‐way analysis of variance (ANOVA) followed by Student's *t*‐test, two‐tailed, α = 0.05, after confirmation of normal distribution and homogeneity of variances. Symbols indicate significance: * *p* ≤ 0.05; ** *p* ≤ 0.01. Analyses were performed using GraphPad Prism 6 (GraphPad Software Inc.).

Mitochondria are essential organelles for parasites' metabolism, and instability of their membrane potential can lead to parasite death [27]. Changes in mitochondrial membrane potential are closely linked to mitochondrial dysfunction during apoptosis [28]. To investigate whether the I06 extract affects mitochondrial function, we evaluated the Δ*Ψ*_m_ of *L*. (*L*.) *amazonensis* promastigotes using the fluorescent probe rhodamine 123. Promastigotes were treated with I06 extract (1 µg/mL) for 24 h, followed by incubation with rhodamine 123. Compared with untreated controls, cells exposed to I06 extract showed a marked reduction in fluorescence intensity, indicating Δ*Ψ*_m_ despolarization (Figure 3A). These results demonstrate that I06 extract induces significant mitochondrial dysfunction by depolarizing the mitochondrial membrane potential in *L*. (*L*.) *amazonensis* promastigotes.

The regulation of reactive oxygen species (ROS) and redox homeostasis is crucial for cellular survival, and disruption of mitochondrial membrane function can disrupt this balance. In apoptosis, dysfunctional mitochondria often lead to ROS overproduction, resulting in oxidative stress and cellular damage [29]. To assess whether I06 extract influences ROS generation, promastigotes were treated with the extract (1 µg/mL) for 15, 30, or 60 min, and intracellular ROS levels were measured using the 2’,7’‐dichlorodihydrofluorescein diacetate (H_2_DCF‐DA) probe. A significant increase in ROS production was detected after 15 min (2.1‐fold) and 30 min (1.7‐fold) compared with untreated controls (Figure 3B). Interestingly, ROS levels returned to baseline after 60 min of exposure. In contrast, pentamidine induced a significant rise in ROS only after 60 min of treatment (Figure 3B). Together, these findings suggest that I06 extract rapidly disrupts mitochondrial function in *L. (L.) amazonensis*, leading to Δψm depolarization and transient ROS overproduction. This oxidative imbalance may contribute to apoptosis‐like cell death in the parasite.

### 2.4 I06 Extract Treatment Protects Mice in Experimental *L*. (*L*.) *amazonensis* Infection

**FIGURE 4:**
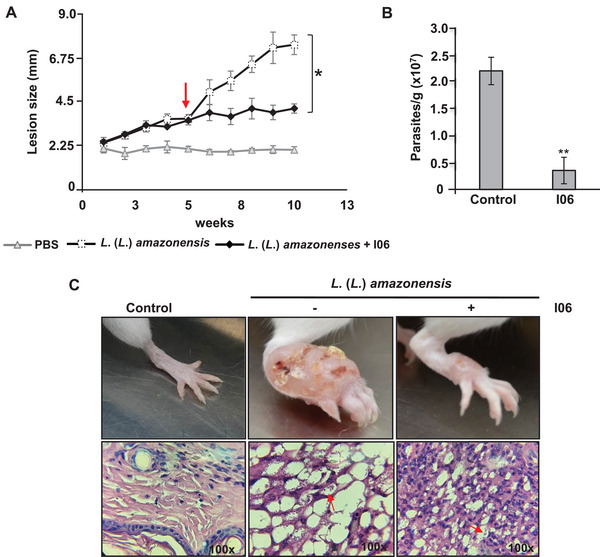
**Secondary metabolite (SM) I06 reduced lesion caused by *L*. (*L*.) *amazonensis* infection in Balb/c mice**. BALB/c mice were infected subcutaneously in the right paw with *L. (L.) amazonensis* promastigotes (1 × 10⁶). After 5 weeks of infection, treatment with SM I06 (3 mg/kg) was initiated (indicated by the red arrow) and administered every 48 h. Paw edema was measured weekly. (A) Size of the paw (mm) was measured using a pachymeter. (B) Load of parasites was determined by a limiting dilution assay. (C) Representative images of mouse paws from each experimental group (top panel) and histological sections of infected paws after 10 weeks of infection (red arrows indicate vacuolated macrophages heavily loaded with amastigotes). Data are presented as mean ± SD (*n* = 6 animals per group). Statistical analysis was performed using one‐way analysis of variance (ANOVA) followed by Student's *t*‐test, two‐tailed, α = 0.05, after confirmation of normal distribution and homogeneity of variances. Symbols indicate significance: * *p* ≤ 0.05; ** *p* ≤ 0.01. Analyses were performed using GraphPad Prism 6 (GraphPad Software Inc.).

We first evaluated the potential hemolytic activity of the I06 extract to assess its safety profile. A hemolysis assay was performed using erythrocytes exposed to different extract concentrations, and no hemolysis was detected at any concentration tested (Figure S1), indicating that the extract was non‐toxic to red blood cells. To investigate its therapeutic potential, BALB/c mice were infected with *L*. (*L*.) *amazonensis*, and lesion development was monitored weekly. Treatment with I06 extract (3 mg/kg, intraperitoneally) began 5 weeks post‐infection. Mice receiving I06 extract showed a significant reduction in lesion size compared to the control group (infected mice treated with phosphate‐buffered saline [PBS]) (Figure 4A). After 10 weeks, animals were euthanized, and parasite load in the paws was quantified by limiting dilution. The I06‐treated group exhibited a fourfold reduction in parasite numbers per gram of tissue compared to the PBS‐treated controls (Figure 4B). These results indicate that I06 extract significantly limits both lesion progression and parasite proliferation in infected mice.

Macroscopic analysis also revealed visibly reduced paw swelling in I06‐treated mice relative to untreated controls. Histological examination of paw tissue confirmed these findings: mice treated with I06 extract showed moderate inflammatory infiltration in the dermis, with macrophages displaying minimal parasitic burden after 10 weeks (Figure 4C, lower panel). In contrast, untreated mice presented extensive inflammatory infiltration, characterized by vacuolated macrophages heavily loaded with amastigotes (Figure 4C, lower panel). Collectively, these data demonstrate that I06 extract treatment exerts a protective effect in experimental *L*. (*L*.) *amazonensis* infection, reducing both parasite burden and associated inflammatory response at the lesion site. Further studies are warranted to elucidate its mechanism of action and to evaluate its safety and efficacy in clinical settings.

Administration of pentamidine is well known to cause hepatotoxicity and nephrotoxicity [30]. In this study, we assessed potential hepatic and renal toxicity of the treatments by measuring serum transaminases and creatinine levels. No statistically significant differences were observed between the control group and mice treated with the I06 extract (Figure S2). Histological analysis of liver and kidney tissues further confirmed the absence of morphological alterations in I06‐treated mice. Although this in vivo model did not include a direct comparison with pentamidine, the absence of biochemical and histological alterations suggests a favorable safety profile for I06. Considering the nephrotoxicity and hepatotoxicity effects of pentamidine reported in the literature, these findings are encouraging for the continued exploration of I06 as a potentially safer therapeutic alternative (Figures S2 and S3). Nevertheless, in mice infected with *L. (L.) amazonensis* and treated with 3 mg/kg of the I06 organic extract, histological analysis revealed mild renal changes (Figure S3). These include a slight increase in glomerular size, vascular congestion, and mild degeneration of proximal and distal convoluted tubules. Importantly, no interstitial alterations were detected, indicating that the observation changes were limited and of low severity.

### 2.5 Liquid Chromatography‐Mass Spectrometry‐based Metabolomics Analysis

The liquid chromatography‐mass spectrometry (LC‐MS) analysis of the extracts I06, I26, I41, and I75, derived from *A. fumigatus*, revealed a diverse chemical profile. An exploratory extraction using ethyl acetate as the sole solvent initially led to the annotation of 14 compounds. To refine the analysis, a more stringent selection was performed based on the number of biological replicates and prior evidence of leishmanicidal activity. As a result, five compounds were selected and are highlighted in Table 3. Sample I41 was included in this analysis as a control, showing no leishmanicidal activity. I06 extract presented Gelomulide N and Tryprostatin B; I26 showed the presence of Gelomulide N, cyclo(Phe‐Leu), Cyclo(Pro‐Val), and Fumigaclavine C; I41 included Cyclo(Pro‐Val) and Fumigaclavine C; and I75 was found to contain cyclo(Phe‐Leu) and Fumigaclavine C. Table 3 displays the Global Natural Products Social (GNPS) compound annotations for each extract, along with key details for each annotation.

**TABLE 3:** Global Natural Products Social (GNPS) dereplication compound annotations for the extracts I06, I26, I41, and I75.

Class	Annotation	*m/z*	Sample	Structure	Error ppm	Cosine	GNPS Library Quality
**Diterpenoids**	Gelomulide N	415.213	I06	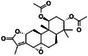	2.26	0.93	Gold
			I26				
**Peptide alkaloids|Small peptides**	Cyclo(Phe‐Leu)	261.1605	I26	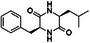	0.76	0.91	Bronze
			I75				
**Small peptides**	Tryprostatin B	352.201	I06	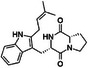	3.97	0.83	Bronze
	Cyclo(Pro‐Val)	197.128	I26	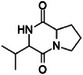	3.04	0.78	Gold
			I41				
**Tryptophan alkaloids**	Fumigaclavine C	367.240	I26	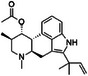	6.39	0.71	Gold
			I75				
			I41				

**FIGURE 5:**
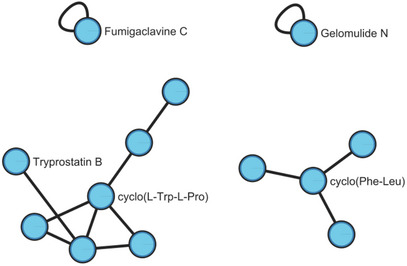
Cytoscape molecular network illustrating the compounds annotated for the extracts.

Figure 5 illustrates the molecular network with the compound annotation. Each cluster displays a compound with an annotation alongside other compounds with similar chemical structures, represented by unannotated nodes.

In the present study, a diverse collection of fungi was isolated from composters located in the SPZPF area, a region characterized by secondary Atlantic Forest vegetation. Composting plant material from the Atlantic Forest fosters a unique microbiome due to the remarkable biodiversity of this biome [31]. The Atlantic Forest is a biodiversity hotspot, home to numerous endemic plant species. Plants from such ecosystems are known to harbor a rich diversity of fungi capable of producing bioactive molecules with potential therapeutic applications against neglected tropical diseases [32]. These fungi have the potential to produce unique and chemically diverse SMs, which may be screened for activity against a variety of pathogens, including parasites responsible for tropical diseases such as leishmaniasis [33]. In this study, we identified an *A. fumigatus* strain isolated from composted plant material collected in the Atlantic Forest, an ecologically complex environment where intense microbial competition drives diverse production of SMs. Under these natural conditions, *A. fumigatus* likely produces a repertoire of metabolites distinct from those typically expressed during mammalian infection. Our findings highlight the potential of this environmental *A. fumigatus* isolate as a promising source of bioactive compounds relevant for the therapeutic of neglected tropical diseases [34].

We specifically investigated the leishmanicidal activity of SM I06, a set of SMs produced by this *A. fumigatus*. Our SM I06 exhibits potent in vitro activity against both the promastigote and amastigote forms of the parasite *L. (L.) amazonensis* (Table 1 and Figure 1A,B), with an IC_50_ value below 1 µg/mL and low cytotoxicity toward 3T3 cells (IC_50_ = 49.72 µg/mL). This high selectivity suggests promising therapeutic potential. In vivo assays further supported these findings, showing significant reductions in lesion size and parasite load (Figure 4), consistent with previous reports of leishmanicidal activity from compounds derived from *Aspergillus* spp. [15].

Compounds with antiparasitic activity often induce notable changes in parasite morphology and structure, which are early indicators of parasite death [35]. These alterations can disrupt osmotic balance [36] and, in some cases, involve the inhibition of serine protease, leading to deformation of the flagellar pouch and the appearance of intracellular vesicles [37]. In our study, SM I06 treatment caused marked morphological changes in *L. amazonensis* consistent with apoptosis (Figure 2C), including mitochondrial alterations, chromatin condensation, and nuclear fragmentation [38, 39]. We also observed increased ROS levels (Figure 3B) and loss of mitochondrial membrane potential (Figure 3A), both recognized as hallmarks of apoptosis in protozoan parasites, which possess a single large mitochondrion whose dysfunction is a critical marker of cell death [40]. These findings align with reports on serine protease inhibitors as potential antiparasitic agents [41].

Current leishmaniasis treatments, including pentamidine and amphotericin B, are limited by nephrotoxicity and hepatotoxicity [42, 43], which are commonly monitored through changes in ALT/AST levels [44]. Moreover, resistance and toxicity issues continue to hinder the effectiveness of available therapies.[45] SM I06 was effective against *L. (L.) amazonensis* without signs of nephrotoxicity or hepatotoxicity (Figures S2 and S3) and did not induce hemolysis in mouse erythrocytes (Figure S1), which is a common issue with some drugs [46], further supporting its potential as a selective, low‐toxicity therapeutic candidate.

Metabolomics profiling revealed that Gelomulide N was present in both I06 and I26 extracts. Although its therapeutic applications are scarcely described, prior studies on Gelomulide A have demonstrated leishmanicidal activity with IC_50_ values below 20 µg/mL [47, 48]. Tryprostatin B, also detected in I06, has documented activity against amastigote‐like forms of *L. amazonensis* and intracellular amastigotes of *Leishmania infantum* [49] and was likewise identified in *A. fumigatus* extract by Doyle et al. [50] Fumigaclavine C, an alkaloid from *Aspergillus* spp., is known for antibacterial effects [51, 52] but has not been evaluated for antileishmanial potential. Cyclo(Phe‐Leu) and cyclo(Pro‐Val) have no reported activity against *Leishmania*. Notably, both Fumigaclavine C and cyclo(Pro‐Val) were present in I41, a non‐active extract, suggesting they are unlikely to be responsible for the observed antileishmanicidal effects. In summary, I06 contained both Gelomulide N and Tryprostatin B, compounds previously reported for their potential antileishmanial activity. While our metabolomic analysis, these findings justify further efforts toward purification, structural characterization, and activity‐guided fractionation to identify the specific constituents responsible for the observed biological effects.

## 3 Conclusions

In conclusion, we report the potential of fungi isolated from composters in the Atlantic Forest region as a valuable source of bioactive molecules with therapeutic potential against neglected tropical diseases such as leishmaniasis. Among the crude extracts identified, SM I06 demonstrated potent activity against both the promastigote and amastigote forms of *L. (L.) amazonensis*, indicating its promise as a potential therapeutic agent for leishmaniasis. The study also provides insights into the mechanism of action of antiparasitic compounds, underscoring the role of mitochondrial alterations as a marker of cell death in protozoan parasites. Notably, SM I06 significantly reduced parasitic load and tissue injury in infected animals, with no evidence of nephrotoxicity or hepatotoxicity, supporting its therapeutic potential and biosafety profile. Although this work focused on the screening of crude extracts, future studies will aim to isolate and characterize the specific compound(s) responsible for the observed antileishmanial effects. Given the biosafety concerns associated with large‐scale cultivation of *A. fumigatus*, chemical synthesis of the active compound(s) is planned to enable safe and scalable production for preclinical development. Importantly, the literature is rich in evidence demonstrating the ability of *A. fumigatus* to produce a wide variety of SMs with diverse biological activities, reinforcing its relevance as a natural source of pharmacologically active compounds. Together, these findings highlight SM I06 as a selective and promising candidate for the treatment of leishmaniasis and emphasize the importance of exploring microbial diversity in complex ecosystems such as the Atlantic Forest for drug discovery.

## 4 Experimental

### 4.1 Fungal Strains and Culture Conditions

Seventy‐one fungal isolates (60 filamentous fungi and 11 yeast) were obtained from the microbial cultures collection of the SPZPF (Brazil), in collaboration with Dr. Suzan Pantaroto Vasconcellos from Federal University of São Paulo (UNIFESP, Brazil). The isolation of these fungal strains was previously described by our research group in the study conducted by Lima et al. [53] Briefly, two cultivation systems were established in August 2012, using soil and lignocellulosic residue samples from the Atlantic Rainforest as the primary inoculum sources to maximize fungal recovery. The soil samples were collected from areas within the SPZPF. To enhance fungal growth, these samples, composed of forest soil, tree bark, and leaf debris, were supplemented with sugarcane filter cake (a lignocellulosic biomass) kindly provided by a local sugar and ethanol refinery. The enrichment systems were incubated for 40 days. After this period, 10 g of each cultivation system (in triplicate) were collected in sterile 50 mL plastic tubes, suspended in sterile distilled water, and serially diluted. Aliquots of 100 µL were spread onto Petri dishes containing yeast malt extract agar (YMA) and potato dextrose agar (Oxoid, UK) and incubated at 28 ± 1°C for 1–4 weeks. Pure cultures were selected based on macro‐ and micromorphological features. The fungal strains were preserved on YMA using two methods: cryopreservation at –80°C in 10% glycerol and by the Castellani method [54]. For experimental procedures, isolates were cultivated in malt extract agar (MEA; 13 g/L malt extract, 5.5 g/L peptone, 0.5 g/L yeast extract, pH 4.7–5.0) at 28–30°C for 7 days under agitation. Long‐term conservation was maintained by the Castellani method and storage at 4°C.

### 4.2 SMs Extraction

To assess the production of fungal SMs, the strains were initially cultivated on malt extract agar plates for 7 days. Subsequently, young mycelial fragments (6 mm agar) were transferred to Erlenmeyer flasks containing 500 mL of fresh MEA broth and incubated at 28°C for 7 days under constant agitation at 200 rpm. All fermentation and extraction procedures were performed in triplicate to ensure reproducibility.

At the end of the fermentation period, SMs were extracted using ethyl acetate (analytical grade) as the solvent. The culture broth was first filtered to remove fungal biomass, and 100 mL of ethyl acetate was added to each Erlenmeyer flask. The mixtures were vigorously shaken for 10 min and then transferred to separating funnels to allow phase separation. The organic (ethyl acetate) phase was collected, and 1 g of NaCl was added as a demulsifier. Subsequently, 3 g of anhydrous sodium sulfate was added to remove any residual moisture from the organic phase. The solvent was then evaporated at 50°C to obtain the dried crude extract. The dried extracts were re‐dissolved in ethyl acetate and filtered through 0.22 µm pore‐size membranes prior to bioactivity assays. All extracts were stored at –20°C until further use.

### 4.3 Animal Use and Ethics Statement

Inbred male BALB/c mice, 6–8 weeks old, were purchased from Centro de Desenvolvimento de Modelos Experimentais (UNIFESP, São Paulo, Brazil). Animals were maintained in pathogen‐free conditions, at a temperature of 23–24°C with a light/dark cycle of 12 h and were provided with food and water ad libitum. Animal experimentation was approved by the Ethics Committee on the use of animals at the Federal University of São Paulo (CEUA 9882111116). Animals were handled according to the Brazilian National Council for Animal Experimentation Control guidelines.

### 4.4 Parasite Strains and Culture Conditions

*L. (L.) amazonensis* strain MHOM/BR/1973/M2269 was generously provided by Dr. Clara Lucia Barbieri (Department of Microbiology, Immunology and Parasitology of UNIFESP, Brazil). The promastigotes were cultured in medium 199 (Gibco—Life Technologies) supplemented with 4.2 mM HEPES, 4.2 mM sodium bicarbonate, 1 mM adenine, 5 µg/mL hemin (bovine type I) (Sigma‐Aldrich), and 10% fetal bovine serum (Gibco—Life Technologies). The culture was maintained at 26°C until the logarithmic phase.

### 4.5 ITS Region Sequencing

Fungal DNA was amplified by polymerase chain reaction (PCR) assay according to Pryce [55] using the ITS regions (ITS1 5’‐TCCGTAGGTGAACCTGCGG‐3’ and ITS4 5’‐TCCTCCGCTTATTGATATGC‐3’), CMD5 gene (5’‐CCGAGTACAAGGAGGCCTTC‐3’), and CMD6 gene (5’‐CCGATAGAGGTCATAACGTGG‐3’) sequence‐specific primers. All PCR‐amplified products were sequenced at the Human Genome Studies Center of the University of São Paulo (Brazil) by automated dye termination sequencing. Each PCR‐amplified product was purified with QIAquick PCR Purification Kit (Qiagen, USA) and sequenced using a 16‐capillary 3730 DNA Analyzer (Applied Biosystems). The BigDye—Terminator V3.1 cycle sequencing kit (Thermo Fisher Scientific) was used with protocols supplied by the manufacturer. PCR‐amplified products were sequenced in a forward and reverse direction using the ITS1 primer and the ITS4 primer, respectively. Sequences were visualized and edited using Chromas Version 1.45 [http://www.technelysium.com.au/chromas.html] and DNAStar (Lasergene, Inc.).

### 4.6 In Vitro Activity Against Different Morphological Stages of *Leishmania*

Promastigote forms: To evaluate the activity of *L*. (*L*.) *amazonensis* in promastigote forms (stationary growth phase), a serial dilution of SMs was prepared, ranging from 80 to 2.5 µg/mL, 2 × 10^6^ parasites/mL was added, and maintained at 26°C for 48 h. The viability of promastigotes was checked by resazurin solution [56] (PrestoBlueTM—Life Technologies) and analyzed by spectrofluorometer at 560 nm excitation and 590 nm emission (Biotek—Instruments, INC., Winooski, VT ‐USA).

Amastigote forms: To evaluate the activity of *L*. (*L*.) *amazonensis* in amastigote forms, 2 × 10^5^ monocytic cells THP‐1 (ATCC TIB‐202) were seeded in coverslips (13 mm). THP‐1 cells were treated with 50 ng/mL phorbol 12‐myristate 13‐acetate (PMA, Sigma) at 37°C in 5% CO_2_ incubator for 24 h to achieve differentiation into adherent, non‐dividing macrophages. After activation by PMA, cells were washed and incubated with complete RPMI medium containing stationary phase *L. amazonensis* promastigotes (2 × 10^6^ parasites/mL) at a macrophage/promastigote ratio of 1:10. After 2 h incubation at 37°C, non‐internalized promastigotes were removed by 2–3 successive washes with RPMI. SMs extract (IC_50_: I06–1 µg/mL), positive control (10 µg/mL pentamidine) were then added to the cultures. Cultures were incubated at 37°C for 48 h. Cells were then washed with PBS and stained with hematoxylin‐eosin (HE). Amastigote forms were counted in 100 cells.

Bone marrow‐derived macrophages were produced as previously described by Zamboni and Rabinovitch [57] with modifications. Approximately 1 × 10^6^ macrophages were added to 24‐well plates containing glass cover slips and medium plus 10% FCS at 37°C in a 5% CO_2_ atmosphere. *L. (L.) amazonensis* promastigotes were added in a proportion of ten parasites to one phagocytic cell. After 2 h, the non‐phagocytosed parasites were removed by washing, and the co‐cultures were maintained under the conditions described above for 24 h. The glass cover slips were then washed with PBS and stained with HE (Panótico Rapido LB; Laborclin, Paraná, Brazil). An average of 100 cells was counted in several microscopic fields to determine the phagocytic index (PI).

### 4.7 Apoptosis Assay

Binding of Annexin V‐FITC/PI to cells was assessed using the Dead Cell Apoptosis Kit (ThermoFisher Scientific, Waltham, MA, USA) according to the manufacturer's instructions. Briefly, promastigotes of *L*. (*L*.) *amazonensis* in logarithmic growth phase were incubated with SMs and pentamidine (10 µg/mL) for 24 h at 26°C. Parasites were harvested and washed three times with cold PBS. Samples were resuspended in 100 µL binding buffer, and 5 µL of FITC Annexin V and 5 µL PI were added. Parasites were incubated for 15 min at room temperature in the dark. The samples were then analyzed on a flow cytometer (FACScalibur—Becton Dickinson, New Jersey, USA), and a minimum of 10 000 cells per condition were analyzed in triplicate. Data acquired were analyzed in the FlowJo software, version 9.7.6 (Tree Star).

### 4.8 Mitochondrial Membrane Potential Assay (Δ*Ψ*_m_)

Mitochondrial membrane potential (Δ*ψ*_m_) was measured using the Rhodamine 123 assay (Thermo Fisher Scientific) according to Chazotte [58] with modifications. Briefly, promastigotes of *L*. (*L*.) amazonensis (1 × 10^6^) were treated with SM for 24 or 48 h. Promastigotes were collected and centrifuged at 400 × *g* for 5 min at room temperature. Supernatant was then carefully removed, and the cells were incubated with Rhodamine 123 (10 µL/mL) for 15 min at 37°C in a CO_2_ incubator. Subsequently, the stained cells were washed twice and resuspended in 500 µL of assay buffer for analysis by flow cytometry in FACScalibur (Becton Dickinson, New Jersey, USA), using FlowJo software, version 9.7.6 (Tree Star). A total of 10 000 events were analyzed per sample.

### 4.9 ROS Assay

Intracellular ROS accumulation was measured using the fluorescent probe H_2_DCF‐DA as described by Gupta [59] with some modifications. Briefly, 1 × 10^7^ *L. (L.) amazonensis* promastigotes were incubated in PBS buffer containing SM (I06 1 µg/mL (IC_50_) for 15, 30, or 60 min at 28°C. As a positive control, we used parasites incubated with 100 µM H_2_O_2_. After this treatment, parasites were washed and incubated in the presence of 10 µM H_2_DCF‐DA (Sigma‐Aldrich) for 45 min at 28°C in the dark. After incubation, the parasites were washed again with PBS buffer and analyzed by flow cytometry using a FACScalibur (Becton‐Dickinson, New Jersey, USA). Fluorescence was measured at 517–527 nm, and the data were analyzed using FlowJo software, version 9.7.6 (Tree Star). A total of 10,000 events were analyzed per sample.

### 4.10 Determination of Cellular Morphology in *L. (L.) amazonensis* Promastigotes

*L. amazonensis* promastigotes (1 × 10^6^ cells/mL) were treated with SMs and incubated at 22 ± 2°C for 4, 24, or 48 h. The cells were then harvested, resuspended in PBS (pH 7.4), and observed under an optical microscope (Zeiss Imager A2 Axio—Camera AxioCam MRc Zeiss). The cellular morphology was recorded through AxioVision 4.8 software, and images were processed using Adobe Photoshop 5.5 (Adobe Systems, Inc., Mountain View, CA, USA) software.

### 4.11 Cytotoxicity Assay

The in vitro cytotoxicity of the SMs samples was evaluated using the 3‐[4,5‐dimethylthiazol‐2‐yl]‐2,5‐diphenyltetrazolium bromide (MTT) method. NIH/3T3 cells (1 × 10^4^) were seeded in a 96‐well plate and incubated with different concentrations of SMs for 24 h at 37°C, 5% CO_2_. Cells were washed and subsequently incubated with MTT (Sigma Aldrich) (0.5 mg/mL) reagent at 37°C, 5% CO_2_, for 2 h. The medium was discarded, and 100 µL of cold isopropanol was added to solubilize formazan crystals, and the absorbance was measured by a microplate spectrophotometer (Biotek‐Instruments, INC., Winooski, USA) at 570 nm. The experiment was repeated three times.

### 4.12 *L. (L.) amazonensis* Experimental Infection

Male BALB/c mice (*n* = 6) were subcutaneously inoculated with 1 × 10^6^ *L*. (*L*.) *amazonensis* promastigotes in the right footpad. After 5 weeks of infection, animals were treated with SM (3 mg/kg of body weight) every 48 h. SM was resuspended in 50 µL of sterile and endotoxin‐free PBS. Animals were checked weekly to monitor the presence of palpable edemas by monitoring the induration diameter, as a control inoculum was used with PBS. The parasite burden was evaluated in the paws after 10 weeks of infection. The paws were aseptically removed from euthanized mice and individually homogenized in M199 medium. The number of parasites was determined using a previously described limiting dilution method [60]. For histological analysis, the paws and organs (kidney and liver) were collected and fixed in 10% neutral buffered formalin. The samples were mounted in glass slides and stained with HE. The quantities of inflammatory cells were obtained from sections stained with HE in 10 fields.

### 4.13 Kidney and Liver Monitoring Assays

Serum concentrations of creatinine and transaminases (AST/ALT) were determined in BALB/c mice by spectrophotometric identification in a semiautomatic biochemical analyzer (Cobas Mira‐Roche Diagnostic System) using sets of commercial reagents (Bioclin/Biosystems and Labtest).

### 4.14 Hemolytic Activity Assay

Different concentrations of SM (0, 1, 2, and 4 µg/mL) were solubilized in 950 µL of saline solution. Then, 50 µL of defibrinated mouse erythrocytes were added for incubation at 37°C for 30 min. After centrifugation (4000 × *g*, 1 min), the presence or absence of hemolysis was visually observed. PBS was used as a negative control, and H_2_O served as a positive control.

### 4.15 LC‐MS Conditions

The methodology consisted of using the equipment ultra‐high‐performance LC (UHPLC)‐MS (QTOF Bruker), which comprises a liquid chromatograph Nexera (USA), equipped with an electrospray source (ESI), with a resolution of 30 000, operating in positive ionization mode, with an ion transfer time of 70 µs and prepulse of 5 µs. Mass range *m/z* 50–1200 was selected, AutoMS mode, with collision energy varying from 20 to 65 eV according to *m/z* 50–700, keeping the energy constant at 65 eV for mass values above *m/z* 700. For each cycle, a maximum of five precursor ions was established. The parameters used to operate the equipment were: capillary 4500 V, nebulizing gas (nitrogen) 4 bars, drying gas (nitrogen) 9.0 L/min, and source temperature 200°C. For internal calibration of the system, a 10 nM sodium formate solution in iso‐propanol/water (1:1 v/v) was used. A Kinetex C18 analytical column (100 × 2.1 mm, 2.6 µm) (Phenomenex, USA) was used for chromatographic separation, maintained at 50°C, with a flow rate of 0.35 mL/min. The mobile phase (A) consisted of deionized water, while phase (B) consisted of acetonitrile, both HPLC grade, containing 20 mM formic acid as an additive. Initially, isocratic elution of 0–2 min was applied in 15% of (B), with a subsequent elution gradient of 2–12 min from 15% to 95% of (B), and again isocratic elution of 12–17 min at 95% from (B). 5 µL of the sample was injected in each analysis.

### 4.16 UHPLC‐tandem MS Processing for Molecular Networking Analysis

The MZMine program was used to verify the chromatograms and mass spectra of the suggested products resulting from the biotransformation of coffee residues. Raw mass spectra data were converted from *.d* to .*mzmL* format with MSConvert (ProteoWizard Version: 3.0), followed by submission to the GNPS Molecular Networking platform server using WinSCP software [61]. Data was filtered by removing all tandem MS (MS/MS) fragment ions within ±17 Da of the precursor *m/z*. The MS/MS spectra were window filtered, choosing only the top six fragment ions in the ±50 Da window across the spectrum. The precursor ion mass tolerance was set to 0.02 Da, and an MS/MS fragment ion tolerance of 0.6 Da. A network was then created where the edges were filtered to have a cosine score above 0.5 and more than four corresponding peaks, allowing greater reliability of results. Furthermore, edges between two nodes were retained in the network if and only if each node appeared in the top 10 most similar nodes. Finally, the maximum size of a molecular family was set to 100, and the lowest‐scoring edges were removed from the molecular families until the molecular family size was below this threshold. The spectra in the network were then searched against the GNPS spectral libraries for dereplication. The library spectra were filtered in the same way as the input data. All matches maintained between network spectra and library spectra were required to have a score above 0.6 and at least four matching peaks [62]. Cytoscape 3.10.1 was used to manipulate the molecular network generated from the samples.

### 4.17 Statistical Analysis

Analysis of variance followed by Student's t‐test was applied to evaluate the effect of the crude extract exposition. The level of significance was set to 5%. All statistical tests were performed using Prism 6 (GraphPad Software Inc.).

## Author Contributions

**Juliana C. P. Calado**: writing – original draft, methodology, investigation, formal analysis, visualization, and software. **Marina V. Navarro**: methodology, investigation, and formal analysis. **Alison F. A. Chaves**: writing – review and editing and formal analysis. **Isis Casula**: investigation and formal analysis. **Patricia L. Ramos**: resources. **João Batista Cruz**: resources. **Anderson M. Rodrigues**: investigation and formal analysis. **Keith D. L. Lira**: investigation and formal analysis. **Fernando L. A. Fonseca**: investigation and formal analysis. **Patricia Xander**: investigation, formal analysis, software, and supervision. **Suzan P. Vasconcellos**: resources, methodology, supervision, and conceptualization. **Wagner L. Batista**: writing – review and editing, conceptualization, resources, supervision, project administration, and funding acquisition.

## Conflicts of Interest

The authors declare no conflicts of interest.

## Supporting information




**Supporting File 1**: cbdv70436‐sup‐0001‐SuppMat.pdf

## Data Availability

Data are available on request from the corresponding author.

## References

[1] A. M. Calvo , R. A. Wilson , J. W. Bok , and N. P. Keller , “Relationship Between Secondary Metabolism and Fungal Development,” Microbiology and Molecular Biology Reviews 66, no. 3 (2002): 447–459. 12208999 10.1128/MMBR.66.3.447-459.2002 PMC120793

[2] N. P. Keller , “Fungal Secondary Metabolism: Regulation, Function and Drug Discovery,” Nature Reviews Microbiology 17, no. 3 (2019): 167–180. 30531948 10.1038/s41579-018-0121-1 PMC6381595

[3] F. Alberti , G. D. Foster , and A. M. Bailey , “Natural Products From Filamentous Fungi and Production by Heterologous Expression,” Applied Microbiology and Biotechnology 101, no. 2 (2017): 493–500. 27966047 10.1007/s00253-016-8034-2 PMC5219032

[4] A. M. Calvo and J. W. Cary , “Association of Fungal Secondary Metabolism and Sclerotial Biology,” Frontiers in Microbiology 6 (2015): 62. 25762985 10.3389/fmicb.2015.00062 PMC4329819

[5] J. H. Yu and N. Keller , “Regulation of Secondary Metabolism in Filamentous Fungi,” Annual Review of Phytopathology 43 (2005): 437–458. 10.1146/annurev.phyto.43.040204.140214 16078891

[6] A. Osbourn , “Secondary Metabolic Gene Clusters: Evolutionary Toolkits for Chemical Innovation,” Trends in Genetics 26, no. 10 (2010): 449–457. 20739089 10.1016/j.tig.2010.07.001

[7] F. Y. Lim and N. P. Keller , “Spatial and Temporal Control of Fungal Natural Product Synthesis,” Natural Product Reports 31, no. 10 (2014): 1277–1286. 25142354 10.1039/c4np00083h PMC4162804

[8] S. A. Kalinina , A. Jagels , B. Cramer , R. Geisen , and H. U. Humpf , “Influence of Environmental Factors on the Production of Penitrems A–F by *Penicillium crustosum* ,” Toxins (Basel) 9, no. 7 (2017): 210. 28671569 10.3390/toxins9070210 PMC5535157

[9] R. Subramani and W. Aalbersberg , “Culturable Rare Actinomycetes: Diversity, Isolation and Marine Natural Product Discovery,” Applied Microbiology and Biotechnology 97, no. 21 (2013): 9291–9321. 24057404 10.1007/s00253-013-5229-7

[10] L. Yan , J. Zhu , X. Zhao , J. Shi , C. Jiang , and D. Shao , “Beneficial Effects of Endophytic Fungi Colonization on Plants,” Applied Microbiology and Biotechnology 103, no. 8 (2019): 3327–3340. 30847542 10.1007/s00253-019-09713-2

[11] A. Rokas , J. H. Wisecaver , and A. L. Lind , “The Birth, Evolution and Death of Metabolic Gene Clusters in Fungi,” Nature Reviews Microbiology 16, no. 12 (2018): 731–744. 30194403 10.1038/s41579-018-0075-3

[12] V. Martinho , L. M. Dos Santos Lima , C. A. Barros , et al., “Enzymatic Potential and Biosurfactant Production by Endophytic Fungi From Mangrove Forest in Southeastern Brazil,” AMB Express 9, no. 1 (2019): 130. 31428885 10.1186/s13568-019-0850-1 PMC6702500

[13] S. Monggoot , T. Pichaitam , C. Tanapichatsakul , and P. Pripdeevech , “Antibacterial Potential of Secondary Metabolites Produced by *Aspergillus* sp., an Endophyte of *Mitrephora wangii* ,” Archives of Microbiology 200, no. 6 (2018): 951–959. 29610939 10.1007/s00203-018-1511-5

[14] J. F. Rojas‐Aedo , C. Gil‐Durán , A. Del‐Cid , et al., “The Biosynthetic Gene Cluster for Andrastin A in *Penicillium roqueforti* ,” Frontiers in Microbiology 8 (2017): 813. 28529508 10.3389/fmicb.2017.00813 PMC5418334

[15] I. P. da Silva , E. Brissow , L. C. Kellner Filho , et al., “Bioactive Compounds of Aspergillus Terreus—F7, an Endophytic Fungus From *Hyptis suaveolens* (L.) Poit,” World Journal of Microbiology and Biotechnology 33, no. 3 (2017): 62. 28243983 10.1007/s11274-017-2228-3

[16] L. W. Mendes and S. M. Tsai , “Distinct Taxonomic and Functional Composition of Soil Microbiomes Along the Gradient Forest‐restinga‐mangrove in Southeastern Brazil,” Antonie Van Leeuwenhoek 111, no. 1 (2018): 101–114. 28831604 10.1007/s10482-017-0931-6

[17] R. F. Dall'Agnol , C. Bournaud , S. M. de Faria , G. Béna , L. Moulin , and M. Hungria , “Genetic Diversity of Symbiotic Paraburkholderia Species Isolated From Nodules of *Mimosa pudica* (L.) and *Phaseolus vulgaris* (L.) Grown in Soils of the Brazilian Atlantic Forest (Mata Atlântica)",” Fems Microbiology Ecology 93, no. 4 (2017). 10.1093/femsec/fix027 28334155

[18] N. Myers , R. A. Mittermeier , C. G. Mittermeier , G. A. da Fonseca , and J. Kent , “Biodiversity Hotspots for Conservation Priorities,” Nature 403, no. 6772 (2000): 853–858. 10706275 10.1038/35002501

[19] P. L. Ramos , M. Y. Kondo , S. M. B. Santos , et al., “A Tropical Composting Operation Unit at São Paulo Zoo as a Source of Bacterial Proteolytic Enzymes,” Applied Biochemistry and Biotechnology 187, no. 1 (2019): 282–297. 29936594 10.1007/s12010-018-2810-7

[20] L. P. Antunes , L. F. Martins , R. V. Pereira , et al., “Microbial Community Structure and Dynamics in Thermophilic Composting Viewed Through Metagenomics and Metatranscriptomics,” Scientific Reports 6 (2016): 38915. 27941956 10.1038/srep38915 PMC5150989

[21] Ó. J. Sánchez , D. A. Ospina , and S. Montoya , “Compost Supplementation With Nutrients and Microorganisms in Composting Process,” Waste Management 69 (2017): 136–153. 28823698 10.1016/j.wasman.2017.08.012

[22] World Health Organization (WHO) . Leishmaniasis . (2025), https://www.who.int/health‐topics/leishmaniasis/the‐global‐epidemiology‐of‐leishmaniasis.

[23] I. Kevric , M. A. Cappel , and J. H. Keeling , “New World and Old World *Leishmania* Infections: A Practical Review,” Dermatologic Clinics 33, no. 3 (2015): 579–593. 26143433 10.1016/j.det.2015.03.018

[24] W. S. Koko , I. S. Al Nasr , T. A. Khan , R. Schobert , and B. Biersack , “An Update on Natural Antileishmanial Treatment Options From Plants, Fungi and Algae,” Chemistry and Biodiversity 19, no. 1 (2022): e202100542. 34822224 10.1002/cbdv.202100542

[25] S. Gannavaram and A. Debrabant , “Programmed Cell Death in Leishmania: Biochemical Evidence and Role in Parasite Infectivity,” Frontiers in Cellular and Infection Microbiology 10 (2012): 2:95. 10.3389/fcimb.2012.00095 PMC3417670 22919685

[26] G. Kroemer , L. Galluzzi , P. Vandenabeele , et al., “Classification of Cell Death: Recommendations of the Nomenclature Committee on Cell Death 2009,” Cell Death and Differentiation 16, no. 1 (2009): 3–11. 18846107 10.1038/cdd.2008.150 PMC2744427

[27] I. H. Tomasco and E. P. Lessa , “The Evolution of Mitochondrial Genomes in Subterranean Caviomorph Rodents: Adaptation Against a Background of Purifying Selection,” Molecular Phylogenetics and Evolution 61, no. 1 (2011): 64–70. 21723951 10.1016/j.ympev.2011.06.014

[28] E. Gottlieb , S. M. Armour , M. H. Harris , and C. B. Thompson , “Mitochondrial Membrane Potential Regulates Matrix Configuration and Cytochrome c Release During Apoptosis,” Cell Death and Differentiation 10, no. 6 (2003): 709–717. 12761579 10.1038/sj.cdd.4401231

[29] L. Galluzzi , O. Kepp , and G. Kroemer , “Mitochondria: Master Regulators of Danger Signalling,” Nature Reviews Molecular Cell Biology 13, no. 12 (2012): 780–788. 23175281 10.1038/nrm3479

[30] Y.‐L. Cao , Z.‐G. Tian , F. Wang , et al., “Characteristics and Clinical Outcome of Nonsteroidal Anti‐inflammatory Drug‐Induced Acute Hepato‐nephrotoxicity Among Chinese Patients,” World Journal of Gastroenterology 20, no. 38 (2014): 13956. 25320533 10.3748/wjg.v20.i38.13956 PMC4194579

[31] L. A. Rodrigues , D. K. A. da Silva , and A. M. Yano‐Melo , “Arbuscular Mycorrhizal Fungal Assemblages in Conservation Unit of Atlantic Forest Areas Under Native Vegetation and Natural Regeneration,” Microbial Ecology 82, no. 1 (2021): 122–134. 33410937 10.1007/s00248-020-01653-z

[32] S. C. Heard , G. Wu , and J. M. Winter , “Antifungal Natural Products,” Current Opinion in Biotechnology 69 (2021): 232–241. 33640596 10.1016/j.copbio.2021.02.001

[33] H. R. A. Cardona , T. Q. Froes , B. C. Souza , et al., “Thermal Shift Assays of Marine‐derived Fungal Metabolites From *Aspergillus fischeri* MMERU 23 Against *Leishmania Major* Pteridine Reductase 1 and Molecular Dynamics Studies",” Journal of Biomolecular Structure & Dynamics 40, no. 22 (2022): 11968–11976. 34415221 10.1080/07391102.2021.1966510

[34] L. H. Rosa , V. N. Gonçalves , R. B. Caligiorne , et al., “Leishmanicidal, Trypanocidal, and Cytotoxic Activities of Endophytic Fungi Associated With Bioactive Plants in Brazil,” Brazilian Journal of Microbiology 41, no. 2 (2010): 420–430. 24031513 10.1590/S1517-838220100002000024 PMC3768680

[35] V. Kaplum , J. Cogo , D. P. Sangi , T. Ueda‐Nakamura , A. G. Corrêa , and C. V. Nakamura , “In Vitro and In Vivo Activities of 2,3‐Diarylsubstituted Quinoxaline Derivatives Against *Leishmania amazonensis* ,” Antimicrobial Agents and Chemotherapy 60, no. 6 (2016): 3433–3444. 27001812 10.1128/AAC.02582-15 PMC4879393

[36] P. G. Bray , M. P. Barrett , S. A. Ward , and H. P. de Koning , “Pentamidine Uptake and Resistance in Pathogenic Protozoa: Past, Present and Future,” Trends in Parasitology 19, no. 5 (2003): 232–239. 12763430 10.1016/s1471-4922(03)00069-2

[37] R. E. Silva‐Lopez , J. A. Morgado‐Díaz , M. A. Chávez , and S. Giovanni‐De‐Simone , “Effects of Serine Protease Inhibitors on Viability and Morphology of *Leishmania* (Leishmania) *amazonensis* Promastigotes,” Parasitology Research 101, no. 6 (2007): 1627–1635. 17726617 10.1007/s00436-007-0706-5

[38] C. Paris , P. M. Loiseau , C. Bories , and J. Bréard , “Miltefosine Induces Apoptosis‐Like Death in Leishmania donovani Promastigotes,” Antimicrobial Agents and Chemotherapy 48, no. 3 (2004): 852–859. 14982775 10.1128/AAC.48.3.852-859.2004 PMC353131

[39] J. F. Alzate , A. A. Arias , D. Moreno‐Mateos , A. Álvarez‐Barrientos , and A. Jiménez‐Ruiz , “Mitochondrial Superoxide Mediates Heat‐Induced Apoptotic‐Like Death in *Leishmania Infantum* ,” Molecular and Biochemical Parasitology 152, no. 2 (2007): 192–202. 17300844 10.1016/j.molbiopara.2007.01.006

[40] A. Jiménez‐Ruiz , J. F. Alzate , E. T. Macleod , C. G. Lüder , N. Fasel , and H. Hurd , “Apoptotic Markers in Protozoan Parasites,” Parasites and Vectors 3 (2010): 104. 21062457 10.1186/1756-3305-3-104 PMC2993696

[41] P. A. Machado , M. P. D. Carneiro , A. J. Sousa‐Batista , et al., “Leishmanicidal Therapy Targeted to Parasite Proteases,” Life Sciences 219 (2019): 163–181. 30641084 10.1016/j.lfs.2019.01.015

[42] R. H. Berdichevski , L. B. Luis , L. Crestana , and R. C. Manfro , “Amphotericin B‐related Nephrotoxicity in Low‐Risk Patients,” Brazilian Journal of Infectious Diseases 10, no. 2 (2006): 94–99. 10.1590/s1413-86702006000200005 16878259

[43] M. A. Cunha , A. C. Leão , R. de Cassia Soler , and J. A. Lindoso , “Efficacy and Safety of Liposomal Amphotericin B for the Treatment of Mucosal Leishmaniasis From the New World: A Retrospective Study,” American Journal of Tropical Medicine and Hygiene 93, no. 6 (2015): 1214–1218. 26483120 10.4269/ajtmh.15-0033 PMC4674237

[44] H. Forouzandeh , M. E. Azemi , I. Rashidi , M. Goudarzi , and H. Kalantari , “Study of the Protective Effect of *Teucrium polium* L. Extract on Acetaminophen‐Induced Hepatotoxicity in Mice,” Iranian Journal of Pharmaceutical Research 12, no. 1 (2013): 123–129. 24250580 PMC3813216

[45] S. M. Lee , M. S. Kim , F. Hayat , and D. Shin , “Recent Advances in the Discovery of Novel Antiprotozoal Agents,” Molecules 24, no. 21 (2019): 3886. 31661934 10.3390/molecules24213886 PMC6864685

[46] M. M. Lubran , “Hematologic Side Effects of Drugs,” Annals of Clinical and Laboratory Science 19, no. 2 (1989): 114–121. 2665627

[47] M. Foroogh , J. G. Yadegari , and I. Salimikia , “Antileishmanial Activity of Natural Diterpenoids: A Comprehensive Review,” Current Organic Chemistry 27 (2023): 772–781.

[48] M. Mangisa , D. Kemboi , G. Fouche , et al., “Ethnomedicinal Uses, Phytochemistry and Pharmacological Properties of Suregada Genus: A Review,” Pharmaceuticals 16, no. 10 (2023): 1390. 37895862 10.3390/ph16101390 PMC10610488

[49] L. G. Tunes , V. N. Goncalves , D. N. Bueno , C. L. Zani , L. H. Rosa , and B. B. Cota , “Diketopiperazine Alkaloids Produced by the Endophytic Fungus *Penicillium Citrinum* and Evaluation of Their Antileishmanial Activity",” African Journal of Microbiology Research 13 (2019): 562–567.

[50] S. Doyle , G. W. Jones , and S. K. Dolan , “Dysregulated Gliotoxin Biosynthesis Attenuates the Production of Unrelated Biosynthetic Gene Cluster‐Encoded Metabolites in *Aspergillus fumigatus* ,” Fungal Biology 122 (2018): 214–221. 29551195 10.1016/j.funbio.2017.12.007

[51] L. Shen , L. Zhu , Q. Luo , et al., “Fumigaclavine I, a New Alkaloid Isolated From Endophyte *Aspergillus terreus* ,” Chinese Journal of Natural Medicines 13, no. 12 (2015): 937–941. 26721713 10.1016/S1875-5364(15)30101-1

[52] A. Gupta , V. Meshram , M. Gupta , et al., “Fungal Endophytes: Microfactories of Novel Bioactive Compounds With Therapeutic Interventions; a Comprehensive Review on the Biotechnological Developments in the Field of Fungal Endophytic Biology Over the Last Decade,” Biomolecules 13, no. 7 (2023): 1038. 37509074 10.3390/biom13071038 PMC10377637

[53] L. M. S. Lima , D. N. Okamoto , M. R. Z. Passarini , et al., “Enzymatic Diversity of Filamentous Fungi Isolated From Forest Soil Incremented by Sugar Cane Solid Waste,” Environmental Technology 43, no. 20 (2022): 3037–3046. 33826477 10.1080/09593330.2021.1914179

[54] C. H. De Capriles , S. Mata , and M. Middelveen , “Preservation of Fungi in Water (Castellani): 20 Years,” Mycopathologia 106, no. 2 (1989): 73–79. 2797113 10.1007/BF00437084

[55] T. M. Pryce , S. Palladino , I. D. Kay , and G. W. Coombs , “Rapid Identification of Fungi by Sequencing the ITS1 and ITS2 Regions Using an Automated Capillary Electrophoresis System,” Medical Mycology 41, no. 5 (2003): 369–381. 14653513 10.1080/13693780310001600435

[56] M. J. Corral , E. González , M. Cuquerella , and J. M. Alunda , “Improvement of 96‐well Microplate Assay for Estimation of Cell Growth and Inhibition of *Leishmania* With Alamar Blue,” Journal of Microbiological Methods 94, no. 2 (2013): 111–116. 23707202 10.1016/j.mimet.2013.05.012

[57] D. S. Zamboni and M. Rabinovitch , “Nitric Oxide Partially Controls *Coxiella burnetii* Phase II Infection in Mouse Primary Macrophages,” Infection and Immunity 71, no. 3 (2003): 1225–1233. 12595436 10.1128/IAI.71.3.1225-1233.2003 PMC148841

[58] B. Chazotte , “Labeling Mitochondria With MitoTracker Dyes,” Cold Spring Harbor Protocols 2011, no. 8 (2011): 990–992. 21807856 10.1101/pdb.prot5648

[59] S. Gupta , V. Bhatia , J. J. Wen , Y. Wu , M. H. Huang , and N. J. Garg , “ *Trypanosoma cruzi* Infection Disturbs Mitochondrial Membrane Potential and ROS Production Rate in Cardiomyocytes",” Free Radical Biology and Medicine 47, no. 10 (2009): 1414–1421. 19686837 10.1016/j.freeradbiomed.2009.08.008 PMC2767388

[60] H. C. Lima , J. A. Bleyenberg , and R. G. Titus , “A Simple Method for Quantifying *Leishmania* in Tissues of Infected Animals,” Parasitology Today 13, no. 2 (1997): 80–82. 15275128 10.1016/s0169-4758(96)40010-2

[61] A. T. Aron , E. C. Gentry , K. L. McPhail , et al., “Reproducible Molecular Networking of Untargeted Mass Spectrometry Data Using GNPS,” Nature Protocols 15, no. 6 (2020): 1954–1991. 32405051 10.1038/s41596-020-0317-5

[62] M. Wang , J. J. Carver , V. V. Phelan , et al., “Sharing and Community Curation of Mass Spectrometry Data With Global Natural Products Social Molecular Networking,” Nature Biotechnology 34, no. 8 (2016): 828–837. 10.1038/nbt.3597 PMC5321674 27504778

