# Supplementary material for: Fungal Biodiversity From the Atlantic Forest With Bioactive Metabolites Against Cutaneous Leishmaniasis
Source: Chem Biodivers. 2025 Aug 28;22(12):e01468. doi: 10.1002/cbdv.202501468 (PMC12716003; doi:10.1002/cbdv.202501468)
Supplement: Supplementary file 1 — Supporting File 1: cbdv70436‐sup‐0001‐SuppMat.pdf [file CBDV-22-e01468-s001.pdf]

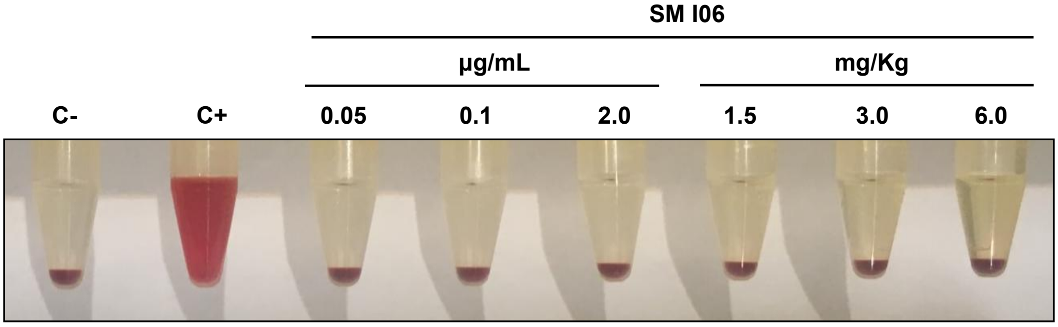


**Supplementary Figure 1S. Hemolysis.** A total of 50 µL of red blood cell were collected from Balb/c in tubes containing heparin and added 500 µL of solution saline (0.9%) with different concentrations of SM I06. $H_2O$ ultrapure was used as positive control. The experiment was performed in triplicate on three independent occasions, and no hemolysis was observed in any replicate. The image shown is representative of the results obtained.

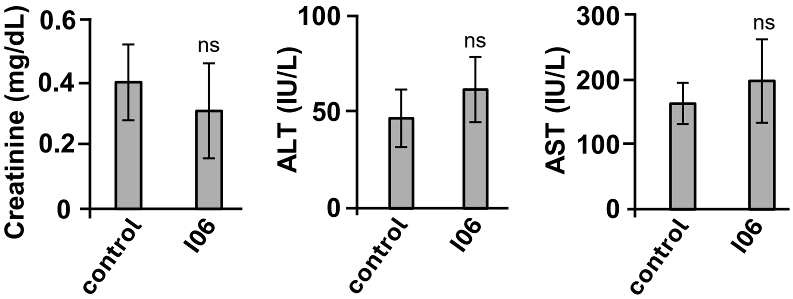


**Supplementary Figure 2S.** The biochemical markers AST, ALT and creatinine were analyzed. After treatment with SM I06 (3 mg/Kg) mice blood Balb/c were collected, and the serum analyzed. Data are presented as mean ± SD (n = 6 animals per group). Statistical analysis was performed using one-way ANOVA followed by Student's *t*-test, two-tailed, $\alpha = 0.05$, after confirmation of normal distribution and homogeneity of variances. "ns" indicates no statistically significant difference. Analyses were performed using GraphPad Prism 6 (GraphPad Software Inc.).

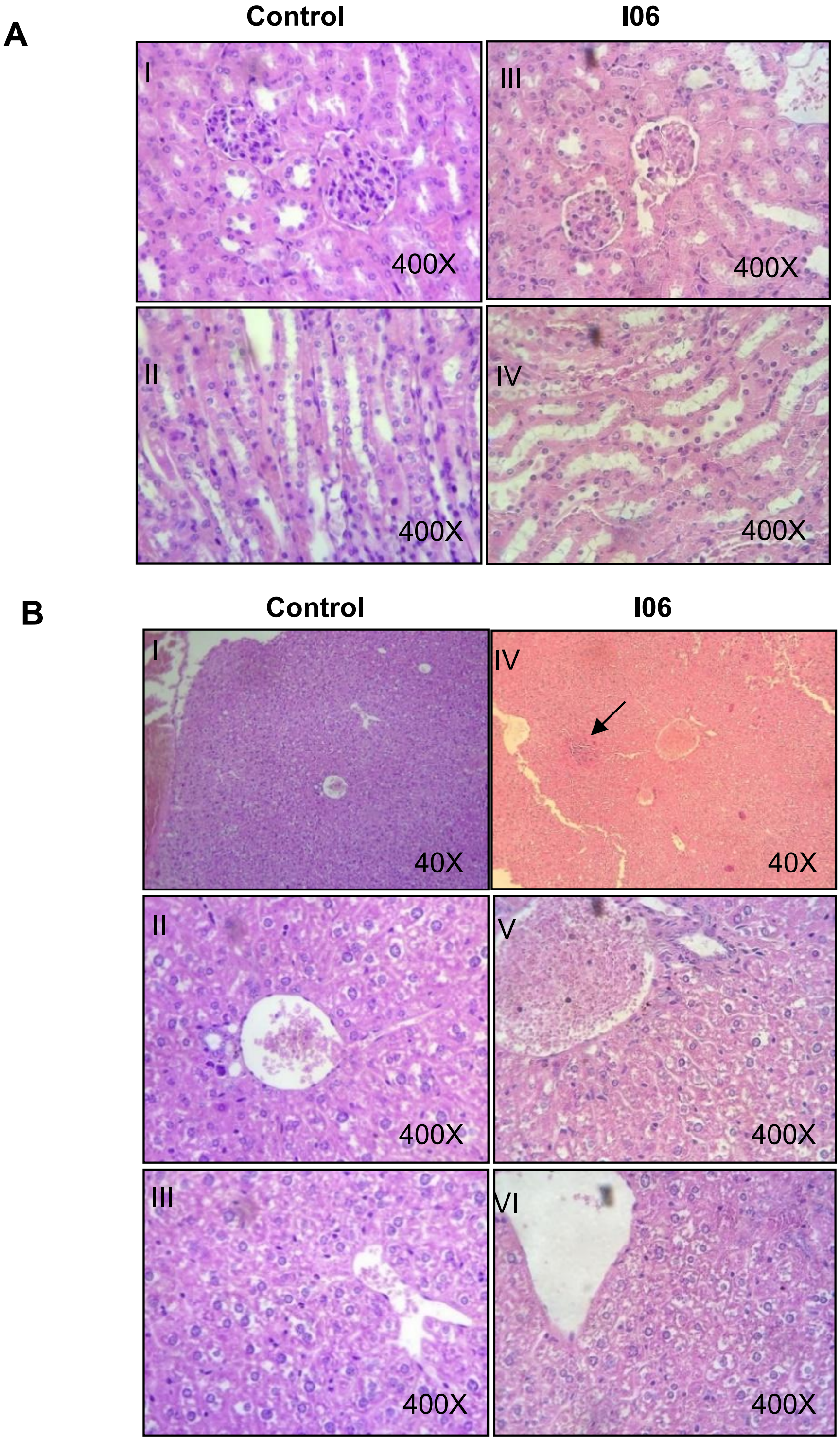


**Supplementary Figure 3S. Kidney and Liver preserved after treatment with SM I06.** (**A**) Images of hitological sections of mice kidneys stained by H&E. (I) Regular glomerular structures and contorted tubules healthy (400x). (II) Henle handle has preserved structure (400x). (III) Glomerular structures regular and contorted tubules with slight tubular degeneration (400X). (IV) Henle handles preserved structure. (**B**) images of hitological sections of mice livers stained by H&E. (I) Lobular structure with discreet disarray with a door and vein space in the center lobular (40x). (II) Door space showing hepatocyte degeneration is periphery (400x). (III) Hepatocytes with mild/moderate diffuse microvacuolar degeneration around vein lobular center (400x). (IV) On the arrow we observed a small group of mononuclear inflammatory cells (40x). (V and VI) Hepatocytes with mild/moderate diffuse microvacuolar degeneration (400X). Images are representative of observations from six animals (n = 6). Statistical analysis was not performed as the figure shows representative histological images.
